# Loss of EP2 Receptor Subtype in Colonic Cells Compromise Epithelial Barrier Integrity by Altering Claudin-4

**DOI:** 10.1371/journal.pone.0113270

**Published:** 2014-11-14

**Authors:** Manigandan Lejeune, France Moreau, Kris Chadee

**Affiliations:** Department of Microbiology, Immunology and Infectious Diseases, University of Calgary, Calgary, Alberta, Canada; Emory University School of Medicine, United States of America

## Abstract

Prostaglandin E_2_ (PGE_2_) is a bioactive lipid mediator that exerts its biological function through interaction with four different subtypes of E-Prostanoid receptor namely EP1, EP2, EP3 and EP4. It has been known that EP2 receptor is differentially over-expressed in the epithelia of inflamed human colonic mucosa. However, the significance of the differential expression in altering epithelial barrier function is not known. In this study, we used Caco-2 cells expressing EP2 receptor, either high (EP2S) or low (EP2A), as a model epithelia and determined the barrier function of these cell monolayers by measuring the trans epithelial resistance (TER). Basal TER of EP2A (but not EP2S) monolayer was significantly lower suggesting a loss of colonic epithelial barrier integrity. In comparison, the TER of wild type Caco-2 was decreased in response to an EP2 receptor specific antagonist (AH-6809) indicating an important role for EP2 receptor in the maintenance of epithelial barrier function. The decrease TER in EP2A monolayer corresponded with a significant loss of the tight junction (TJ) protein claudin-4 without affecting other major TJ proteins. Similarly, EP2 receptor antagonism/siRNA based silencing significantly decreased claudin-4 expression in EP2S cells. Surprisingly, alteration in claudin-4 was not transcriptionally regulated in EP2A cells but rather undergoes increased proteosomal degradation. Moreover, among the TER compromising cytokines examined (IL-8, IL-1β, TNF-α, IFN-γ) only IFN-γ was significantly up regulated in EP2A cells. However, IFN-γ did not significantly decreased claudin-4 expression in Caco-2 cells indicating no role for IFN-γ in degrading claudin-4. We conclude that differential down-regulation of EP2 receptor play a major role in compromising colonic epithelial barrier function by selectively increasing proteosomal degradation of claudin-4.

## Introduction

Prostaglandins E_2_ (PGE_2_) is an important bioactive lipid produced by variety of different tissues including the gastrointestinal (GI) tract [1]–[3] were it modulates both physiological and pathological functions of the gut [4]. The biological activity of PGE_2_ depends on the amount released in the microenvironment and this is regulated by two different isoforms of cyclooxygenase (COX-1 and 2), which metabolize arachidonic acid into PGE_2_ [5], [6]. Following synthesis and release, PGE_2_ signals via four different subtypes of EP receptors namely EP1, EP2, EP3 and EP4 [7]. Evidences suggest that each of these distinct receptors exerts alternate and in some cases opposing signaling cascade [8]. Current thoughts on versatility of PGE_2_ are attributed to EP receptors that it couples and activates. Cell type specific functions of individual EP receptor have been elucidated in the GI tract [4]. More importantly, altered/differential expression of EP receptor subtypes has been reported in various disease conditions in the gut [9]–[13]. However, the role of such alterations in modulating biological functions of PGE_2_ is not clearly understood.

EP2 is an important subtype of EP receptor that is expressed in the GI tract including human colonic epithelium [14]. Studies with various animal models suggest a cytoprotective role for EP2 receptor in the gut. Activation of EP2 receptor prevented ethanol-induced apoptosis of gastric mucosal cells in guinea pigs and radiation-induced apoptosis of jejunal epithelial cells in mice [15], [16]. Interestingly, expression of EP2 receptor is also altered in disease conditions of the gut. In radiation-induced injury of mice, EP2 receptor expression was increased in the large intestine [16] that corresponded with epithelial restitution of the injured tissues. In inflammatory bowel diseases (ulcerative colitis), EP2 receptor up-regulation was apparent in lateral crypt epithelia of colonic mucosa but the significance of this is not clear [14]. At present, nothing is known about the biological relevance of differentially expressed EP2 receptors in colonic epithelial cells. It is not clear if differential EP2 receptor expression alter colonic epithelial monolayer integrity.

The single layer of epithelial cells lining the colonic mucosa forms an important barrier between host and the intestinal lumen. The barrier function of epithelia is greatly determined by tight junction (TJ) that seals intercellular space [17], [18]. TJ is made of 40 different proteins, which consist of occludin, JAM, ZO-1 and members of family of claudins that regulate barrier permeability [19], [20]. Dysregulation of this barrier occurs in disease condition affecting normal cellular environment and results in organ failure [21]. TJ barrier is disrupted by a variety of pathogens including bacteria, virus and parasite as well as by host inflammatory mediators [22]–[27]. Circumstantial evidence suggests lipid mediators of inflammation including PGE_2_ may play a role in altering colonic epithelial barrier function. PGE_2_ apparently plays a dual role in maintaining intestinal mucosal barrier function. For example, it is associated with recovery of barrier integrity in response to hypoxia-induced injury [28] and is also equally implicated in barrier compromising enteric diseases such as inflammatory bowel diseases [29], [30]. One study implicates EP2 receptor activation in altering colonic epithelial TJ barrier integrity [31]. However, in inflamed colonic mucosa EP2 receptor is differentially expressed in the epithelium and the functional significance of such expression (over- *vs* down-regulation) on epithelial TJ barrier function is not known. We hypothesize that differential EP2 receptor expression in colonic epithelial cells can alter barrier function. Therefore, the aim of this study was do understand the effect of altered expression of EP2 receptor on colonic epithelial integrity and to elucidate a role for EP2 receptor in regulating TJ protein expression.

## Materials and Methods

### Materials

Butaprost and AH-6809 (Catalog no: 13740 and 14050) were purchased from Cayman chemical. MG-132 (Catalog no: 474790) was from Calbiochem. Geneticin (G-418) was purchased from Invitrogen. The antibody for EP2 receptor (C-Term) was from Cayman chemical (Catalog no: 101750) and COX-2 was from Santa Cruz (Catalog no:1745). The antibodies for ZO-1, Occludin, Claudin-1, Claudin-2 & Claudin-4 were purchased from Zymed laboratories, Invitrogen (Catalog no: 617300; 711500; 519000; 516100 & 329400, respectively). IFN-γ was from R&D Systems. Twelve well transwell plates (Catalog no: 3460) were from Corning, Costar. All other chemical were purchased from Sigma-Aldrich or otherwise mentioned.

### Caco-2 cells and EP2 receptor transfectants

Caco-2 human colonic cells (ATCC Manassas, VA) were maintained in Modified Eagle's Medium (MEM) supplemented with 10% fetal bovine serum, 100 units/ml penicillin, 100 µg/ml streptomycin sulfate and 20 mM HEPES. They were incubated at 37°C in 5% CO_2_ and passaged once the monolayer reached 90% confluency. To passage cells, monolayer in a T75 flask was rinsed with 5 ml of sterile PBS and incubated with 1 ml of Trypsin-EDTA for 3–8 min. Detached cells were mixed vigorously to avoid clumps and resuspended in media to a final concentration of 1×10^6^cells/ml. To prepare a 12 well transwell plate, 5×10^4^ cells were seeded on the apical side of the membrane containing 500 µl media and the basolateral side was bathed only with 1.5 ml of media. The transwell plates were fed the day after seeding and every alternate day thereafter and used for experiments approximately 8–10 days after seeding. Caco-2 cells grown on transwell plates formed a monolayer with a trans epithelial resistance (TER) of ∼1100 Ω/cm^2^. Caco-2 cells that express either high (EP2S) or low (EP2A) EP2 receptor were developed and characterized in our laboratory as previously described [32]. Briefly, EP2 receptor transfectants along with their vector control were grown in MEM in the presence of G418. Cells were either seeded on transwell or on regular culture plates and were used for experiments approximately 8 days after seeding.

### RNA interference

EP2 siRNA (Smartpool M-005712-00) and control siRNA (D-001210-02) were obtained from Dharmacon, Inc. (Lafayette, CO, USA). For transfection studies using siRNA, sub confluent EP2S cells grown on regular plates were transfected using gene juice (Novagen) and cells collected after an interval of 48 h.

### Western blotting

Protein extracts were prepared from cells by lysing them in a buffer (50 mM Tris HCl, 140 mM EDTA, 30 mM sodium pyrophosphate, 50 mM sodium fluoride and protease inhibitor cocktail tablet) that contains 1% Triton X-100. The protein content of the fractions was estimated using Bradford method and adjusted for a final concentration of 0.5 µg/µl. The protein samples were mixed in an equal volume of 1× sample buffer, incubated at boiling water bath for 10 min and used for blotting. Five µg of total protein were used per well for electrophoreses in 12% SDS-polyacrylamide gels and transferred onto a nitrocellulose membrane (Bio-Rad). Membranes were blocked in 5% skim milk powder in TBS-T (20 mM Tris-HCl, _P_H 7.5, 500 mM NaCl, 0.1% Tween20) for 1 h at room temperature and incubated with appropriate primary antibodies in 1% skim milk-TBS-T at 4°C, overnight. Blots were washed three times with TBS-T and incubated in horseradish peroxidase (HRP) conjugated secondary antibodies in 1% skim milk-TBS-T for 2 h at room temperature. The blots were washed with TBS-T and developed using Immobilon Western Chemiluminescent HRP Substrate (Millipore, Billerica, MA) as per the manufacturer's instructions.

### IFN-γ assay

FN-γ in cell culture supernatant was assayed using Human IFN-γ ELISA Ready-SET-Go! Kit (Catalog no: 88-7316, eBioscience) as per manufacturer's protocol.

### Real time PCR

RNA was extracted from cultured cells using TRIzol reagent (Invitrogen) and quantified. One microgram of RNA was reverse transcribed by using Moloney murine leukemia virus reverse transcriptase (Invitrogen) and Random Primers according to the standard protocol. Eighty nanogram of the cDNA was used for real-time PCR. The primers used were IL-8 (F: CGTGGCTCTCTTGGCAGC, R: TCTTTAGCAC- TCCTTGGCAAAAC); IL-1β (F: ATTGCTCAAGTGTCTGAAGC, R: GTAGTGGTG- GTCGGAGATT); TNF-α (F: TCAGTCAGTGGCCCAGAAGAC, R: GATACCCCTC-ACACTCCCCAT); IFN-γ (F: ACATTCCACAATTGATTTTATTCTTACAACA, R: ACGAGCTTTAAAAGATAGTTCCAAACA); ZO-1 (F: ATGGTGTCCTACCTAATT-CAAATCAT, R: GCCAGCTACAAATATTCCAACATCA); Occludin (F: AAGGTC-AAAGAGAACAGAGCAAGA, R: TATTCCCTGATCCAGTCCTCCTC); Claudin-1 (F: ATGCAATCTTTGTGTCCACCATT, R: ATTCTGTTTCCATACCATGCTGTG); Claudin-2 (F: AAGACTGTGCATCTCATGCC, R: AGCATTGTGACAGCAGTTGG); Claudin-4 (F: TCGTGGGTGCTCTGGGGATGCTT, R: GCGGATGACGTTGTG-AGCGGTC) and actin (F: AGAGGGAAATCGTGCGTGAC, R: CAATAGTGATGA-CCTGGCCGT). Quantitative PCR was performed using SYBR green (Qiagen's quantitect PCR kit) according to manufacture's instruction. The reaction mixture was denatured for 10 min at 95°C and subjected to 45 cycles of three steps PCR consisting of denaturation (95°C for 10 secs), annealing (60°C for 15 sec) and extension (72°C for 20 sec). The amplified products were verified by agarose gel electrophoresis for predicted size. The specificity of amplification was checked by performing a melting curve analysis. mRNA levels of gene of interest (GOI) were expressed as ratio of GOI to actin.

### Statistical analysis

TER data were analyzed by two-way ANOVA followed by Bonferroni posttest for comparison between groups. Unpaired data were analyzed using Student's *t* test. Graphpad Prism version 4.0 (GraphPad Software, San Diego, CA) was used for all statistical analysis. All values are means (with standard error of mean) of three independent experiments unless otherwise indicated. ★,★★,★★★ indicates statistical significance p<0.05, <0.01 and <0.001, respectively.

## Results

### Loss of EP2 receptor decreases TER in Caco-2 colonic epithelial monolayer

**Figure 1:**
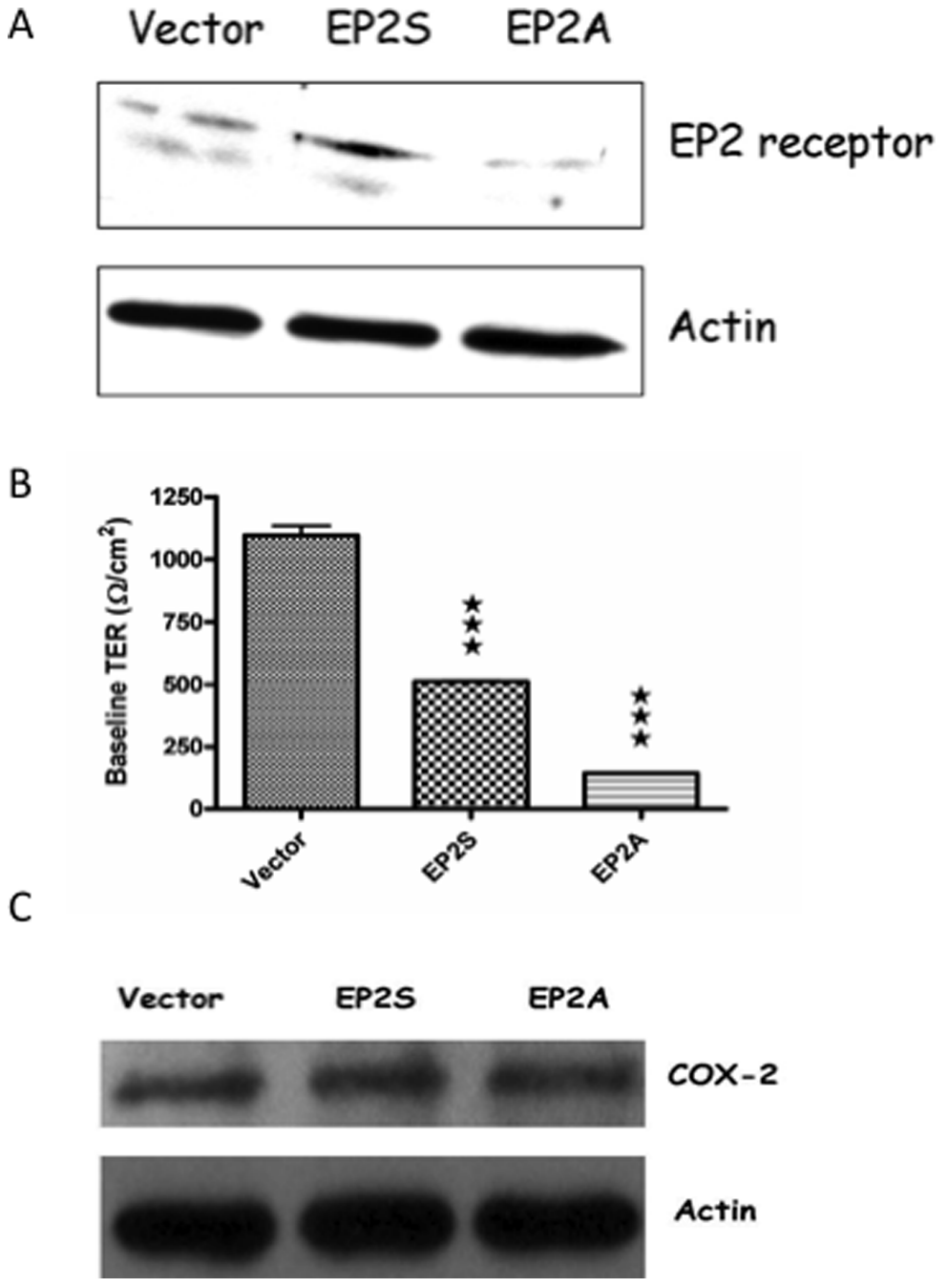
Differential EP2 receptor expression alters TER of Caco-2 epithelial monolayer. (A) A representative immunoblot showing differential EP2 receptor (52 KDa) expression in stable Caco-2 transfectants expressing either high (EP2S) or low (EP2A) EP2 receptors. Vector alone-transfected cells were used as a control. Actin was used as an internal control. (B) The Caco-2 cells (from the above experiments) were seeded on 12-transwell plates and after confluence was reached the TER measured. Basal resistance was measured using a Millicell ERS apparatus and expressed as ohms/cm^2^. TER of each group (n = 6) was statistically compared with that of the vector control. ★★★ p<0.001. (C) A representative immunoblot showing no change in the expression levels of COX-2 in EP2S/A cells and the vector control.

Caco-2 cell lines that stably express either high (EP2S) or low (EP2A) EP2 receptor as well as the vector control were previously developed and established in our laboratory [32]. The level of EP2 receptor expression in the cells was confirmed by western blot. As shown in Figure 1A, EP2 receptor expression was low in EP2A cells as compared to the vector control, whereas EP2S cells showed 4-fold enhanced expression as determined by densitometry. To ascertain if the differential EP2 receptor expression had any effect on epithelial monolayer resistance, we assessed basal TER of the cells grown to confluence on transwell plates. As shown in Figure 1B, control (vector only) monolayer had a baseline TER of 1097±37.94 Ω/cm^2^. However, EP2S monolayer had a TER of 512±4.063 Ω/cm^2^ that was 53% lower than that of the vector control. Even though EP2S monolayer had a significantly (p<0.001) low TER compared to control, the baseline TER was at least 7 times more than the resistance offered by the polyethylene membrane substratum (∼80 Ω/cm^2^) on which the cells were grown. As predicted, EP2A cells constitutively expressed very low TER of 142.3±1.944 Ω/cm^2^, which was 87% lower than that of the vector control (p<0.001). More importantly, TER of EP2A monolayer was very close to that of the substratum. Even though high doses of PGE_2_ have been shown to alter TER in Caco-2 cells [31], none of the transfectants constitutively produce high PGE_2_ [32] and COX-2 expression levels are not altered between the cells (Fig. 1C). From these studies, it is clear that lack of EP2 receptor almost completely abrogated the TER of Caco-2 colonic epithelial monolayer through a mechanism independent of endogenous PGE_2_.

### Pharmacological inhibition of EP2 receptor decreases TER of Caco-2 epithelial monolayer

**Figure 2:**
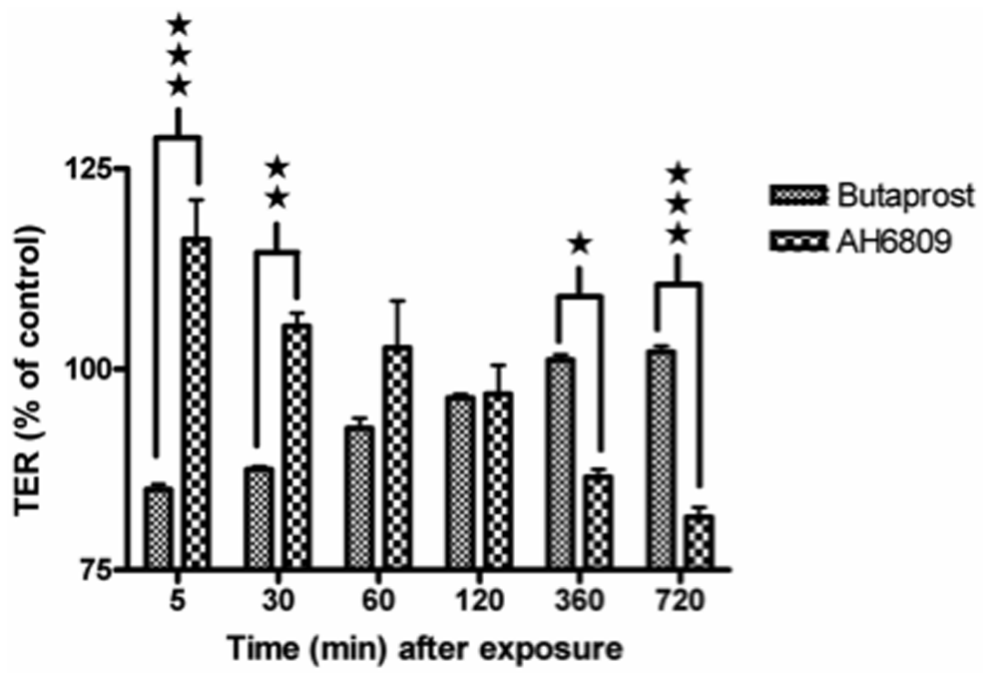
Effect of EP2 receptor agonist and antagonist on TER of Caco-2 epithelial monolayer. Caco-2 monolayer that reached confluence on transwell plate was exposed with either 10 µM of Butaprost (EP2 specific agonist) or 50 µM of AH6809 (EP2 antagonist) on the apical side and TER (Ω/cm^2^) measured at the time indicated for each group, compared to control and depicted as % of control. The mean baseline TER at the start of the experiment was 1025.5±71.5 Ω/cm^2^. ★ p<0.05; ★★ p<0.01 and ★★★ p<0.001.

As EP2A had very low TER, we speculate that wild type Caco-2 monolayer would lose its TER on exposure to an EP2 receptor antagonist (AH6809). A report [31] showed that the EP2 receptor specific agonist, butaprost decreased TER in Caco-2 monolayer. To determine the exact roles of EP2 receptor specific agonist or antagonist on TER, we individually checked the effect of butaprost (10 µM, EP2 agonist) and AH6809 (50 µM) on resistance of Caco-2 monolayer. The concentration of EP2 receptor agonist/antagonist used was determined based on a previous study [32]. TER was measured at time-points over the period of 720 min and compared with non-stimulated controls and expressed as % of control. As shown in Figure 2, butaprost modestly decreased TER (15% decrease at 5 min) and slowly returned back to control levels within 360 min. However, AH6809 increased TER initially (16% increase at 5 min) but dropped latter with a maximum decrease observed at 720 min (18% decrease). Clearly, there is an opposite effect for EP2 receptor agonist and antagonist on TER of Caco-2 monolayer. In fact at 5 min, the difference in mean TER (% of control value) between butaprost and AH6809 treated group (85% *vs* 116%) was highly significant (p<0.001). Similarly at 720 min, the mean TER between the two groups was highly significant (p<0.001) however; the difference (102% vs 82%) was due to reversal in TER compared to that at 5 min. Thus, there is a temporal difference between EP2 receptor agonist and antagonist in decreasing TER. More importantly, prolonged stimulation of Caco-2 monolayer with an EP2 receptor specific antagonist significantly decreased TER.

### EP2A cells constitutively express low claudin-4

**Figure 3:**
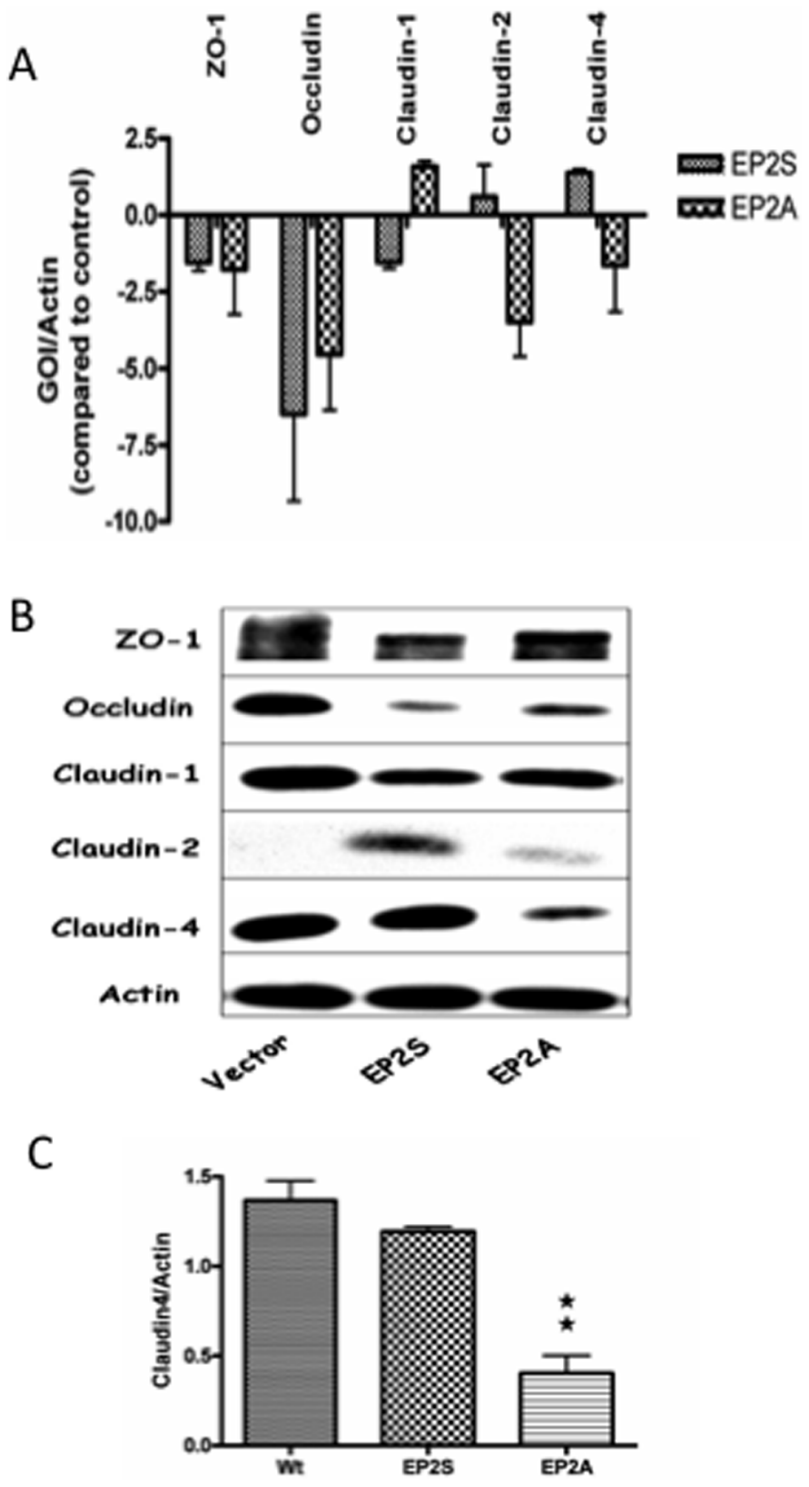
The expression of tight junction mRNA and proteins in Caco-2 EP2 receptor transfectants. (A) The mRNA expression for tight junction proteins (ZO-1, occludin, claudin-1, claudin-2 and claudin-4) in Caco-2 EP2S and EP2A cells was measured by Q-PCR. The expression of gene of interest (GOI) was normalized with the internal control (Actin) and expressed as fold changes with that of the vector control. (B) A representative immunoblot showing constitutive expression of the important tight junction proteins (ZO-1, occludin, claudin-1, claudin-2 and claudin-4) in EP2S, EP2A and vector control cells. Actin used as internal control. (C) Densitometry analysis for claudin-4 expression in EP2S, EP2A and vector control cells. EP2A compared statistically with the vector control. ★★ p<0.01.

Having identified that EP2A monolayer had extremely low TER compared to the vector control or EP2S cells, we correlated the baseline TER of these cell lines (as shown in Fig. 1B) with that of basal expression of TJ proteins. As TER is a measure of TJ integrity and that the presence or absence of TJ proteins is a major determinant of junctional integrity, we screened for expression of the important TJ proteins such as ZO-1, occludin, claudin-1, claudin-2 and claudin-4. Among the TJ proteins the presence of ZO-1, occludin, claudin-1 and claudin-4 can increase TER. In contrast, claudin-2 is a pore forming TJ protein whose presence can decrease TER. Accordingly, the basal mRNA expression of the TJ proteins were analyzed by Q-PCR in EP2A/S cells and compared with that of the vector control (Fig. 3A). As shown, the transcript for occludin was significantly decreased in both EP2A and EP2S cells whereas alterations in the expression for the other TJ proteins remained unchanged. The alterations observed in occludin in both EP2A/S cells did not correlate with differences in TER as depicted in Figure 1B. However, analysis of protein expression by Western blot (Figure 3B) revealed that the vector control express all the TJ proteins except claudin-2 which correlated well with high TER. EP2S cells normally expressed claudin-4 but moderately expressed ZO-1 and claudin-1. Occludin expression was very low but claudin-2 was moderately expressed and correlated with the TER as compared to the vector control. Similar to EP2S, EP2A cells moderately expressed ZO-1 and claudin-1 and had low expression of occludin. Claudin-2 expression was very low. Interestingly, EP2A cells significantly lacked claudin-4 as compared to the vector control or EP2S cells. Densitometry analysis clearly showed a significant decrease in claudin-4 expression (71% decrease; p<0.01) in EP2A cells as compared to the vector control (Fig. 3C). As EP2A monolayer had very low TER and decreased expression of claudin-4 was exclusively observed in these cells, we conclude that loss of EP2 receptor constitutively is related to the decreased expression of claudin-4 in Caco-2 epithelial monolayer.

### Claudin-4 undergoes increased proteosomal degradation in EP2A cell lines

**Figure 4:**
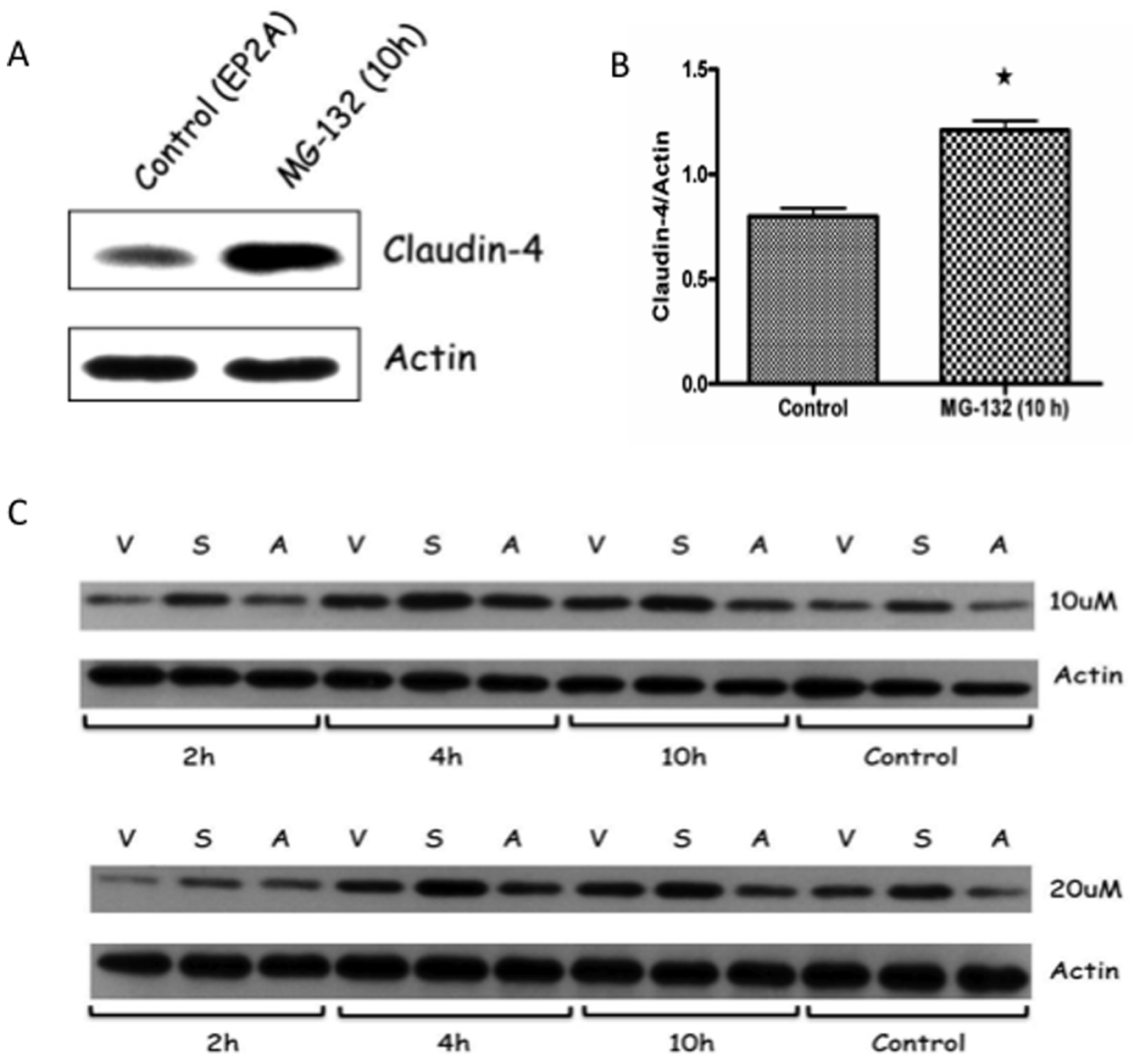
Claudin-4 undergoes increased proteosomal degradation in EP2A cells. (A) EP2A cells grown as monolayer on culture plates were exposed with 20 µM of MG-132 (proteosomal inhibitor) for 10 h. Cells were collected and checked for claudin-4 expression by western blotting. Actin used as internal control protein. (B) The corresponding densitometry analysis for claudin-4 expression, ★ p<0.05. (C) Western blots depicting dose and time response of MG-132 treatment on claudin-4 expression in vector control, EP2S and EP2A cells. Two different doses of MG-132 (10 and 20 µM) exposed for 2, 4 and 10 h time period were studied. Control cells were exposed with only DMSO (diluent for MG-132) for 10 h. V = vector control; S = EP2S; A = EP2A.

It was clear from the western blot analysis that claudin-4 was significantly decreased in EP2A cells. However, mRNA analysis did not show any significant change in claudin-4 expression. This highlights the possibility of claudin-4 protein undergoing increased cellular degradation in EP2A cells. To determine whether loss of claudin-4 was due to increased proteosomal degradation, confluent EP2A monolayer was treated with the proteasome inhibitor (20 µM of MG-132) for 10 h and analyzed by western blotting for claudin-4 expression. As shown in the immunoblot (Fig. 4A), MG-132 treatment significantly rescued claudin-4 from degradation by 51% (p<0.05; Fig. 4B). A dose and time response of MG-132 treatment (two different doses of 10 and 20 µM exposed for 2, 4 and 10 h time period) on claudin-4 expression in vector control, EP2S and EP2A cells showed increased claudin-4 expression in all 3 cell types but was more pronounced in EP2A cells treated with MG-132 as compared to the untreated control (Fig. 4C). These results clearly indicate an exaggerated proteosomal degradation of claudin-4 as the mechanism for the significant lower expression of this important TJ protein in EP2A cells.

### Inhibition or silencing of EP2 receptor decreases expression of claudin-4

**Figure 5:**
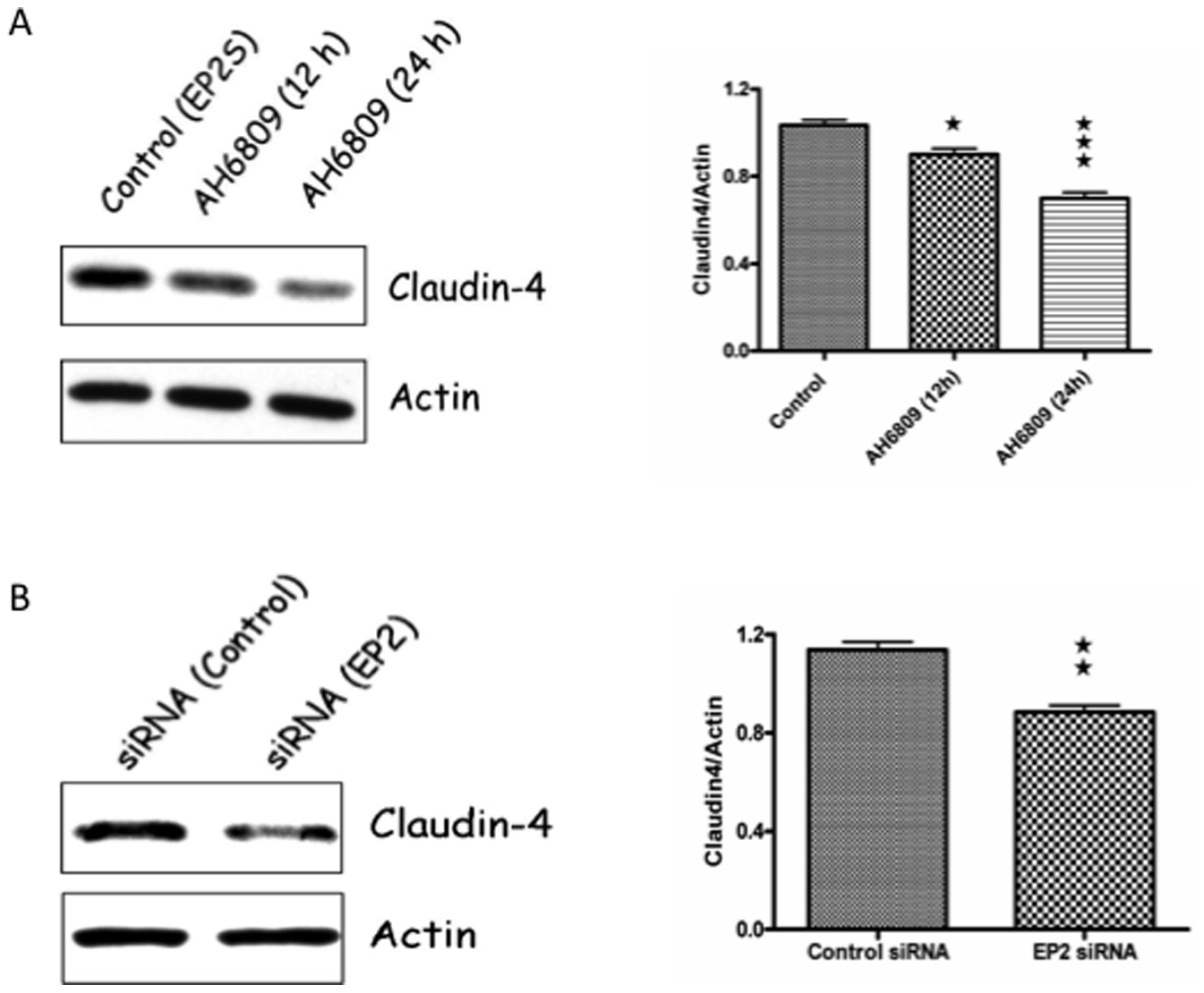
Pharmacological inhibition and/or siRNA silencing of EP2 receptor in EP2S cells decrease claudin-4 expression. (A) EP2S cells that reached confluence on regular culture plates were exposed with 50 µM of AH6809 (EP2 antagonist) for the time indicated and the cell lysate analyzed for claudin-4 expression by western blotting. Actin used as the internal control for the densitometry analysis. (B) EP2S cells seeded on culture plates that reached 60 percent confluence were transfected with either EP2 receptor or control siRNA. Cells collected after 48 h were checked for claudin-4 expression by western blotting and quantified by densitometry analysis for claudin-4 expression. ★ p<0.05; ★★ p<0.01 and ★★★ p<0.001.

The results above suggest that cells lacking EP2 receptor expression was positively correlated with constitutive decrease in claudin-4 expression. To address specificity for this observation, we analyzed whether pharmacological inhibition or RNAi silencing of EP2 receptor could cause a similar decrease in claudin-4 expression. In EP2S cells, the EP2 receptor specific antagonist AH6809 (50 µM) decreased claudin-4 expression by 13 (p<0.05) and 32% (p<0.001) at 12 and 24 h, respectively (Fig. 5A). Similarly, in EP2S cells, siRNA silencing of EP2 receptor decreased claudin-4 expression by 24% within 48 h (p<0.001, Fig. 5B). These studies confirm that either inhibition of EP2 receptor activity or lack of EP2 receptor expression significantly decreased claudin-4.

### EP2A cells up-regulate IFN-γ

**Figure 6:**
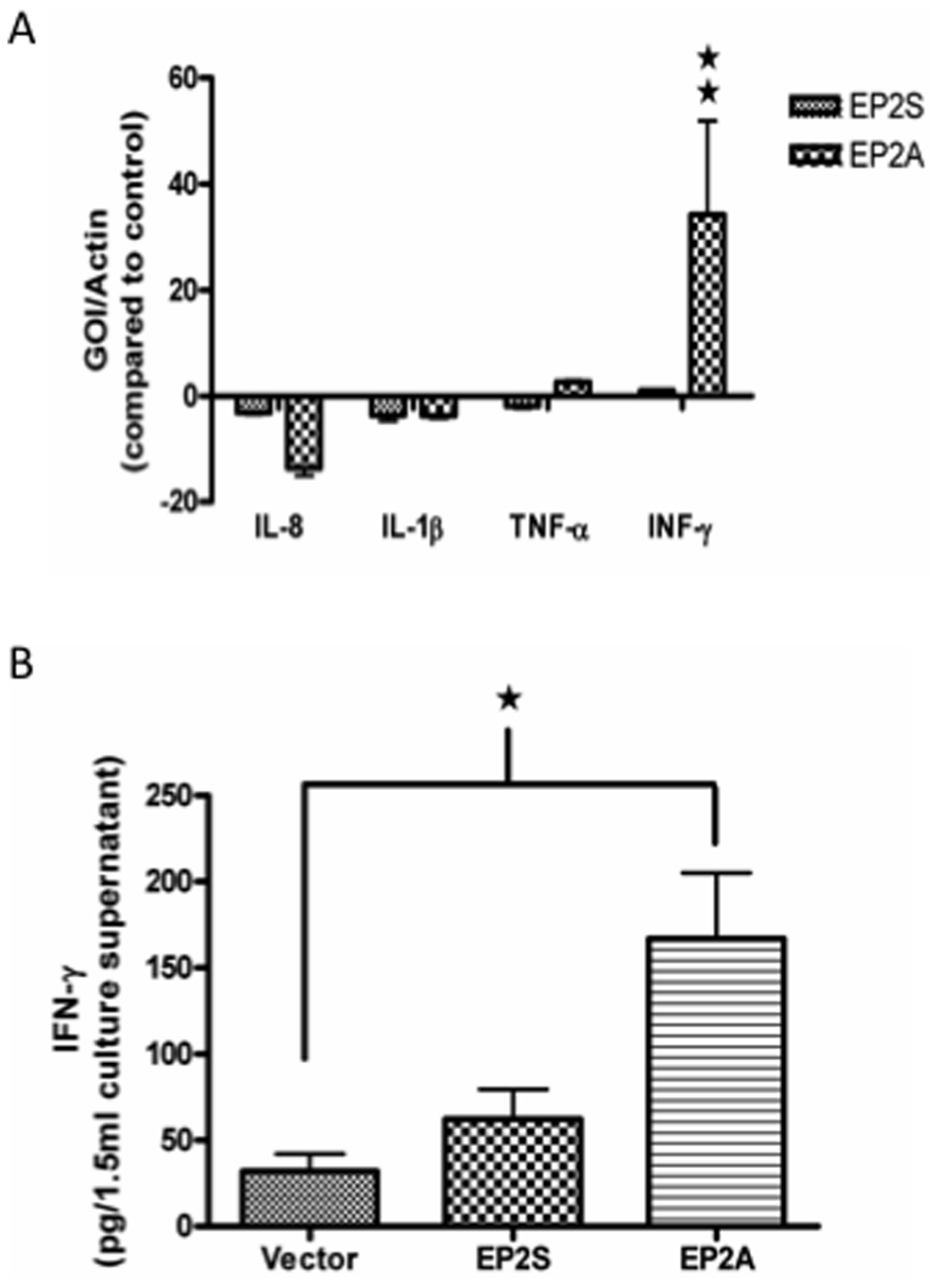
EP2A cells over express IFN-γ. (A) The expression of transcript for cytokines (IL-8, IL-1β, TNF-α and IFN-γ) in Caco-2 EP2S and EP2A cells was measured by Q-PCR. The gene of interest (GOI) was normalized with the internal control (Actin) and depicted as fold changes with that of vector control. IFN-γ expression in EP2A cells was statistically compared with that of vector control. (B) Cell culture supernatant collected from Caco-2 cells (vector control, EP2S and EP2A) were quantified for IFN-γ using eBioscience's Human IFN-γ ELISA Kit. ★ p<0.05; ★★ p<0.01.

From the studies above it is clear that EP2A monolayer expressed low claudin-4 with constitutive low TER. To address why these cells exhibit a lower TER, we explored whether the cells were expressing TER compromising pro-inflammatory cytokines. Accordingly, the basal expression of IL-8, IL-1β, TNF-α and IFN-γ was analyzed by Q-PCR in EP2A/S cells and compared to the vector control. As shown in Figure 6A, only IFN-γ was significantly up regulated (33-fold increase) in EP2A cells as compared to the vector control (p<0.01). Moreover, there was no significant difference in the basal expression of the pro-inflammatory cytokines between EP2S and the vector control clearly implicating selective up-regulation of IFN-γ in EP2A cells. As IFN-γ was a transcriptionally up regulated in EP2A cells, we determined if there was a corresponding increase in IFN-γ secretion. As shown in Figure 6B, the mean value of IFN-γ produced by the vector controls was low (31.98±10.06 pg) similar to the EP2S cells (62.19±17.20 pg). In staked contrast, EP2A cells produced 5 times significantly more IFN-γ (166.9±38.25 pg, p<0.05) than the vector control.

### IFN-γ does not decrease claudin-4 expression in Caco-2 monolayer

**Figure 7:**
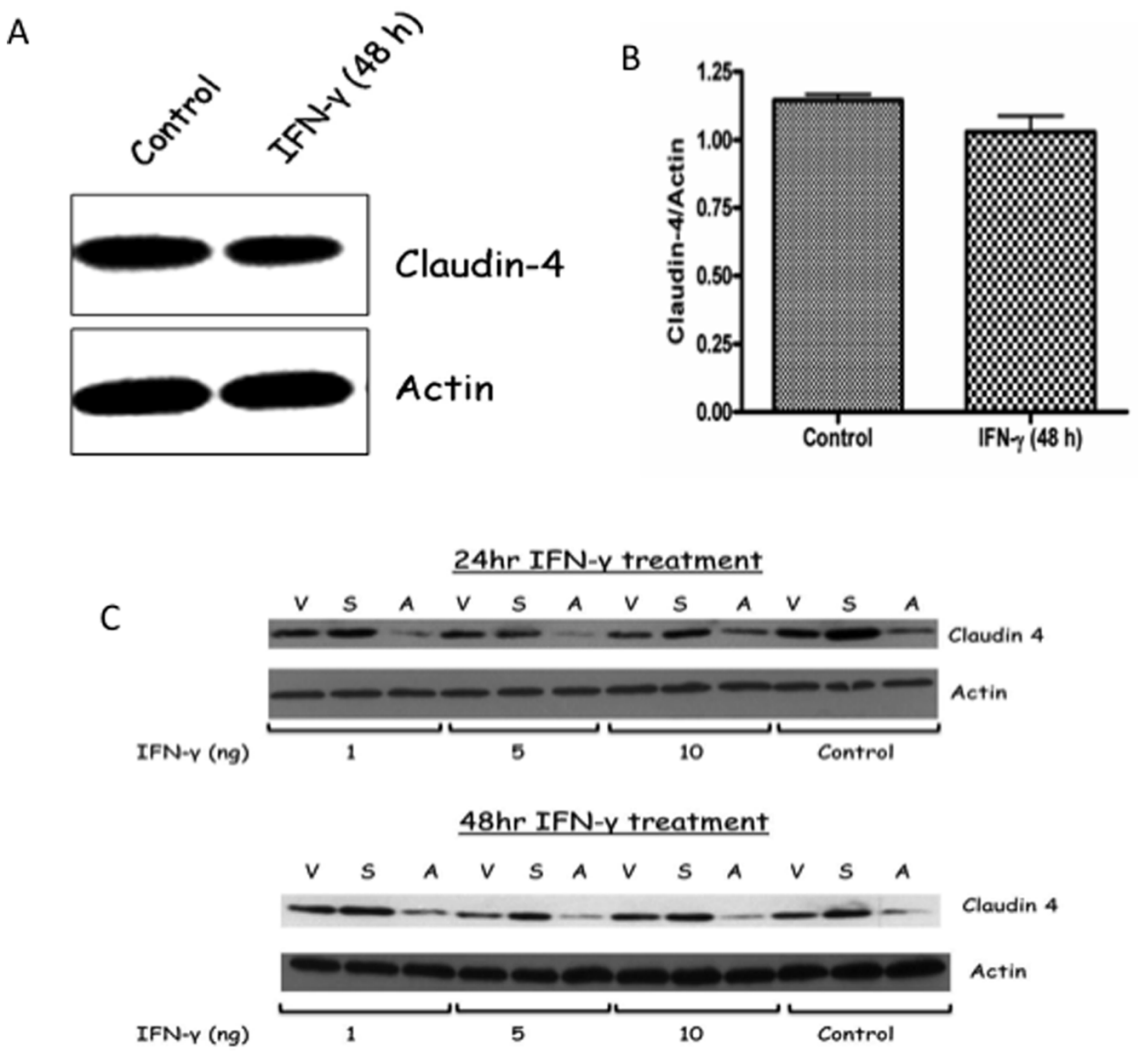
Stimulation of Caco-2 cells with IFN-γ decreases claudin-4 expression. (A) Caco-2 cells grown to confluence on regular culture plates were stimulated with 5 ng/ml of IFN-γ for 48 h. At the end of stimulation, cells were analyzed for claudin-4 expression by western blotting. (B) The corresponding densitometry analysis for claudin-4 expression. (C) Western blots depicting dose and time response of IFN-γ treatment on claudin-4 expression in vector control, EP2S and EP2A cells. Three different doses of IFN-γ (1, 5 and 10 ng) exposed for 24 and 48 h time period were studied and compared with corresponding untreated control cells. V = vector control; S = EP2S; A = EP2A.

As in many transfection studies, up-regulation of IFN-γ is considered as inherent anti-viral response. If decrease in claudin-4 were an effect of anti-viral response, it would negate the role of absence of EP2 receptor in selectively degrading claudin-4. Therefore, we checked the effect of IFN-γ on claudin-4 protein expression. Caco-2 monolayer was stimulated with 5 ng/ml of IFN-γ for 48 h and analyzed by western blot. As shown in Figures 7A–B, prolonged exposure with IFN-γ did not significantly decreased claudin-4 protein expression implicating that factors other than IFN-γ may play role in decreasing claudin-4 in EP2A cells. Even using varied dose and time response of IFN-γ treatment (1, 5 and 10 ng exposed for 24 and 48 h time period), claudin-4 expression in vector control and in EP2S and EP2A cells showed no decrease in claudin-4 expression as compared with untreated control cells (Fig. 7C).

## Discussion

As EP receptor subtypes determine the outcome of biological effect of PGE_2_, considerable attention is now given to study the role of each of these subtypes in gut function. It is known that EP2 receptor is differentially up regulated in colonic epithelium of patients with ulcerative colitis [14]. Although a similar increase in a rodent model of radiation-induced injury associated EP2 receptor with epithelial restitution and repair [16], nothing is known about the role of this receptor in human colonic mucosa. In this study, we use Caco-2 cells as model epithelia to study the effects of either over-expression or down-regulation of EP2 receptor in colonic epithelial integrity. To determine if differential EP2 receptor expression could alter integrity of epithelial monolayer, we measured baseline TER in EP2S/A cells as compared with the vector control. Interestingly, both EP2S/A cells showed low constitutive TER as compared to controls. However, EP2A monolayer had a more pronounced decrease in TER. Although Caco-2 is highly used *in vitro* model for TER/TJ studies, it is still a cancer cell line with elevated expression of COX-2 [33]. Thus the observed decrease in TER of EP2S monolayer could be due to increased stimulation of EP2 receptor by endogenous PGE_2_. In our study, COX-2 expression levels were not altered between EP2S/A and the vector control. This rules out the role of endogenous PGE_2_ in mediating the decrease in TER.

Based on baseline TER of the transfectants, it is difficult to assign an exact role for EP2 receptor in altering barrier integrity. Therefore, we used a pharmacological approach to elucidate the role of EP2 on epithelial integrity. EP2 activation decreased TER initially but steadily increased back to normal levels. EP2 inactivation increased TER initially but dropped down latter. These results suggest that the mechanism by which agonist and antagonist decreases TER are clearly different. While the initial effect of the EP2 receptor agonist on TJ proteins was studied previously [31], we conducted a series of experiments to analyze the fate of the TJ proteins in EP2A cells. Among the TJ proteins studied, claudin-4 expression was constitutively decreased in EP2A cells. Moreover, prolonged exposure with an EP2 receptor antagonist or siRNA based silencing of the EP2 receptor, significantly decreased claudin-4 expression. Claudin-4 is an important TJ protein whose presence at paracellular junction is reported to increase TER [34]. Therefore, the decreased claudin-4 expression in EP2A cells correlated well with extremely low TER in these cells.

We analyzed the cellular mechanism by which lack of EP2 receptor decrease claudin-4 expression. Analysis for mRNA expression shows that the decrease of claudin-4 in EP2A cells is not regulated transcriptionally. However, MG-132 inhibitor study highlights claudin-4 undergoing increased proteosomal degradation. The link between loss of EP2 receptor and selective proteosomal degradation of claudin-4 is intriguing. At present we are not sure why or how claudin-4 is degraded in EP2A cells. We also analyzed whether loss of EP2 receptor up-regulated TER compromising cytokines. IL-8, TNF-α, IL-1β and IFN-γ that are known to break TJ barrier [35], [36] and we investigated if any of these cytokines altered TER in EP2A cells. Special emphasis was placed on IFN-γ as it poses a threat of antiviral-response in transfection studies. Q-PCR analysis showed that only IFN-γ was up regulated and EP2A cells produced and secreted significantly more of IFN-γ as compared to the vector control or EP2S cells. This raises the question whether IFN-γ is responsible for decreasing the expression of claudin-4 in EP2A cells. Previous studies on the effect of IFN-γ on epithelial TJ clearly show depletion of occludin [37]. In our studies we observed depletion of occludin in both EP2A/S cells which corresponded to significant and slight elevation of IFN-γ in these cells. Interestingly, however, loss of claudin-4 was observed exclusively in EP2A cells. Thus to determine if IFN-γ played a role in altering claudin-4 we studied the effect of prolonged exposure of IFN-γ and did not observe a significantly decreased in claudin-4. This corroborates previous studies [36] in T84 colonic epithelial cells that showed no effect of IFN-γ on claudin-4 expression. In summary, our studies show that loss of EP2 receptor in the colonic epithelium can constitutively decrease barrier integrity by altering TJ. Mechanistically, this occurred by selective proteosomal degradation of claudin-4. As our studies were done *in vitro* using colonic epithelial cells, confirmation using animal models will be needed to define the role of EP2 receptors in colonic barrier functions. Nonetheless, our studies highlight the importance of varied biological functions of EP2 receptors in the gut caused by various GI pathologies.

## References

[1] Atay S, Tarnawski AS, Dubois A (2000) Eicosanoids and the stomach. Prostaglandins Other Lipid Mediat 61: 105–124. 10867124 10.1016/s0090-6980(00)00067-8

[2] Mohajer B, Ma TY (2000) Eicosanoids and the small intestine. Prostaglandins Other Lipid Mediat 61: 125–143. 10867125 10.1016/s0090-6980(00)00068-x

[3] Krause W, DuBois RN (2000) Eicosanoids and the large intestine. Prostaglandins Other Lipid Mediat 61: 145–161. 10867126 10.1016/s0090-6980(00)00069-1

[4] Dey I, Lejeune M, Chadee K (2006) Prostaglandin E2 receptor distribution and function in the gastrointestinal tract. Br J Pharmacol 149: 611–623. 17016496 10.1038/sj.bjp.0706923 PMC2014644

[5] O'Banion MK, Winn VD, Young DA (1992) cDNA cloning and functional activity of a glucocorticoid-regulated inflammatory cyclooxygenase. Proc Natl Acad Sci U S A 89: 4888–4892. 1594589 10.1073/pnas.89.11.4888 PMC49193

[6] Xie WL, Chipman JG, Robertson DL, Erikson RL, Simmons DL (1991) Expression of a mitogen-responsive gene encoding prostaglandin synthase is regulated by mRNA splicing. Proc Natl Acad Sci U S A 88: 2692–2696. 1849272 10.1073/pnas.88.7.2692 PMC51304

[7] Narumiya S, Sugimoto Y, Ushikubi F (1999) Prostanoid receptors: structures, properties, and functions. Physiol Rev 79: 1193–1226. 10508233 10.1152/physrev.1999.79.4.1193

[8] Breyer RM, Bagdassarian CK, Myers SA, Breyer MD (2001) Prostanoid receptors: subtypes and signaling. Annu Rev Pharmacol Toxicol 41: 661–690. 11264472 10.1146/annurev.pharmtox.41.1.661

[9] Cosme R, Lublin D, Takafuji V, Lynch K, Roche JK (2000) Prostanoids in human colonic mucosa: effects of inflammation on PGE(2) receptor expression. Hum Immunol 61: 684–696. 10880739 10.1016/s0198-8859(00)00131-2

[10] Jang TJ, Min SK, Bae JD, Jung KH, Lee JI, et al (2004) Expression of cyclooxygenase 2, microsomal prostaglandin E synthase 1, and EP receptors is increased in rat oesophageal squamous cell dysplasia and Barrett's metaplasia induced by duodenal contents reflux. Gut 53: 27–33. 14684572 10.1136/gut.53.1.27 PMC1773937

[11] Shoji Y, Takahashi M, Kitamura T, Watanabe K, Kawamori T, et al (2004) Downregulation of prostaglandin E receptor subtype EP3 during colon cancer development. Gut 53: 1151–1158. 15247185 10.1136/gut.2003.028787 PMC1774140

[12] Sonoshita M, Takaku K, Sasaki N, Sugimoto Y, Ushikubi F, et al (2001) Acceleration of intestinal polyposis through prostaglandin receptor EP2 in Apc(Delta 716) knockout mice. Nat Med 7: 1048–1051. 11533709 10.1038/nm0901-1048

[13] Takafuji VA, Evans A, Lynch KR, Roche JK (2002) PGE(2) receptors and synthesis in human gastric mucosa: perturbation in cancer. Prostaglandins Leukot Essent Fatty Acids 66: 71–81. 12051958 10.1054/plef.2001.0299

[14] Takafuji V, Cosme R, Lublin D, Lynch K, Roche JK (2000) Prostanoid receptors in intestinal epithelium: selective expression, function, and change with inflammation. Prostaglandins Leukot Essent Fatty Acids 63: 223–235. 11049698 10.1054/plef.2000.0144

[15] Hoshino T, Tsutsumi S, Tomisato W, Hwang HJ, Tsuchiya T, et al (2003) Prostaglandin E2 protects gastric mucosal cells from apoptosis via EP2 and EP4 receptor activation. J Biol Chem 278: 12752–12758. 12556459 10.1074/jbc.M212097200

[16] Houchen CW, Sturmoski MA, Anant S, Breyer RM, Stenson WF (2003) Prosurvival and antiapoptotic effects of PGE2 in radiation injury are mediated by EP2 receptor in intestine. Am J Physiol Gastrointest Liver Physiol 284: G490–498. 12431904 10.1152/ajpgi.00240.2002

[17] Farquhar MG, Palade GE (1963) Junctional complexes in various epithelia. J Cell Biol 17: 375–412. 13944428 10.1083/jcb.17.2.375 PMC2106201

[18] Staehelin LA (1973) Further observations on the fine structure of freeze-cleaved tight junctions. J Cell Sci 13: 763–786. 4203962 10.1242/jcs.13.3.763

[19] Denker BM, Nigam SK (1998) Molecular structure and assembly of the tight junction. Am J Physiol 274: F1–9. 9458817 10.1152/ajprenal.1998.274.1.F1

[20] Schneeberger EE, Lynch RD (2004) The tight junction: a multifunctional complex. Am J Physiol Cell Physiol 286: C1213–1228. 15151915 10.1152/ajpcell.00558.2003

[21] Harhaj NS, Antonetti DA (2004) Regulation of tight junctions and loss of barrier function in pathophysiology. Int J Biochem Cell Biol 36: 1206–1237. 15109567 10.1016/j.biocel.2003.08.007

[22] Buret AG, Mitchell K, Muench DG, Scott KG (2002) Giardia lamblia disrupts tight junctional ZO-1 and increases permeability in non-transformed human small intestinal epithelial monolayers: effects of epidermal growth factor. Parasitology 125: 11–19. 12166516 10.1017/s0031182002001853

[23] Leroy A, Lauwaet T, De Bruyne G, Cornelissen M, Mareel M (2000) Entamoeba histolytica disturbs the tight junction complex in human enteric T84 cell layers. FASEB J 14: 1139–1146. 10834936 10.1096/fasebj.14.9.1139

[24] Nava P, Lopez S, Arias CF, Islas S, Gonzalez-Mariscal L (2004) The rotavirus surface protein VP8 modulates the gate and fence function of tight junctions in epithelial cells. J Cell Sci 117: 5509–5519. 15494377 10.1242/jcs.01425

[25] Schulzke JD, Ploeger S, Amasheh M, Fromm A, Zeissig S, et al (2009) Epithelial tight junctions in intestinal inflammation. Ann N Y Acad Sci 1165: 294–300. 19538319 10.1111/j.1749-6632.2009.04062.x

[26] Sonoda N, Furuse M, Sasaki H, Yonemura S, Katahira J, et al (1999) Clostridium perfringens enterotoxin fragment removes specific claudins from tight junction strands: Evidence for direct involvement of claudins in tight junction barrier. J Cell Biol 147: 195–204. 10508866 10.1083/jcb.147.1.195 PMC2164970

[27] Thanabalasuriar A, Koutsouris A, Weflen A, Mimee M, Hecht G, et al (2010) The bacterial virulence factor NleA is required for the disruption of intestinal tight junctions by enteropathogenic Escherichia coli. Cell Microbiol 12: 31–41. 19712078 10.1111/j.1462-5822.2009.01376.x PMC2850276

[28] Moeser AJ, Haskell MM, Shifflett DE, Little D, Schultz BD, et al (2004) ClC-2 chloride secretion mediates prostaglandin-induced recovery of barrier function in ischemia-injured porcine ileum. Gastroenterology 127: 802–815. 15362036 10.1053/j.gastro.2004.06.004

[29] Ahrenstedt O, Hallgren R, Knutson L (1994) Jejunal release of prostaglandin E2 in Crohn's disease: relation to disease activity and first-degree relatives. J Gastroenterol Hepatol 9: 539–543. 7865710 10.1111/j.1440-1746.1994.tb01557.x

[30] Rampton DS, Sladen GE, Youlten LJ (1980) Rectal mucosal prostaglandin E2 release and its relation to disease activity, electrical potential difference, and treatment in ulcerative colitis. Gut 21: 591–596. 7429322 10.1136/gut.21.7.591 PMC1419888

[31] Tanaka MN, Diaz BL, de Souza W, Morgado-Diaz JA (2008) Prostaglandin E2-EP1 and EP2 receptor signaling promotes apical junctional complex disassembly of Caco-2 human colorectal cancer cells. BMC Cell Biol 9: 63. 19055708 10.1186/1471-2121-9-63 PMC2648958

[32] Dey I, Giembycz MA, Chadee K (2009) Prostaglandin E(2) couples through EP(4) prostanoid receptors to induce IL-8 production in human colonic epithelial cell lines. Br J Pharmacol 156: 475–485. 19175605 10.1111/j.1476-5381.2008.00056.x PMC2697677

[33] Tsuji S, Kawano S, Sawaoka H, Takei Y, Kobayashi I, et al (1996) Evidences for involvement of cyclooxygenase-2 in proliferation of two gastrointestinal cancer cell lines. Prostaglandins Leukot Essent Fatty Acids 55: 179–183. 8931116 10.1016/s0952-3278(96)90095-2

[34] Van Itallie C, Rahner C, Anderson JM (2001) Regulated expression of claudin-4 decreases paracellular conductance through a selective decrease in sodium permeability. J Clin Invest 107: 1319–1327. 11375422 10.1172/JCI12464 PMC209303

[35] Al-Sadi RM, Ma TY (2007) IL-1beta causes an increase in intestinal epithelial tight junction permeability. J Immunol 178: 4641–4649. 17372023 10.4049/jimmunol.178.7.4641 PMC3724221

[36] Bruewer M, Luegering A, Kucharzik T, Parkos CA, Madara JL, et al (2003) Proinflammatory cytokines disrupt epithelial barrier function by apoptosis-independent mechanisms. J Immunol 171: 6164–6172. 14634132 10.4049/jimmunol.171.11.6164

[37] Boivin MA, Roy PK, Bradley A, Kennedy JC, Rihani T, et al (2009) Mechanism of interferon-gamma-induced increase in T84 intestinal epithelial tight junction. J Interferon Cytokine Res 29: 45–54. 19128033 10.1089/jir.2008.0128 PMC2988462

